# Exposure of Horses in Israel to West Nile Virus and Usutu Virus

**DOI:** 10.3390/v12101099

**Published:** 2020-09-28

**Authors:** Gili Schvartz, Sharon Tirosh-Levy, Oran Erster, Roni Shenhar, Hadas Levy, Barbara Bazanow, Boris Gelman, Amir Steinman

**Affiliations:** 1 Koret School of Veterinary Medicine, The Hebrew University of Jerusalem, Rehovot 7610001, Israel; giliun@gmail.com (G.S.); sharontirosh@gmail.com (S.T.-L.); rony.shenhar@mail.huji.ac.il (R.S.); hadasylevy@gmail.com (H.L.); 2 Department of Virology, Kimron Veterinary Institute, Bet Dagan 50200, Israel; borisg@moag.gov.il; 3 Central Virology Laboratory, Haim Sheba Medical Center, Ministry of Health, Ramat-Gan 5265601, Israel; Oran.Erster@sheba.health.gov.il; 4 Department of Pathology, Wrocław University of Environmental and Life Sciences, 50-375 Wroclaw, Poland; barbara.bazanow@upwr.edu.pl

**Keywords:** West Nile virus, Usutu virus, horse, Israel

## Abstract

West Nile virus (WNV) and Usutu virus (USUV) are arboviruses transmitted by mosquito vectors. Whereas WNV is endemic in Israel, the Middle East, Europe, and in the Americas, data regarding the prevalence of USUV in the Middle East is limited. While both viruses share similar reservoirs and vectors, exposure of horses in the area to USUV have never been assessed. The aim of this study was to estimate the seroprevalence and co-exposure of WNV and USUV in horses in Israel. A total of 327 serum samples from healthy unvaccinated horses in Israel collected in 2018 were tested for neutralizing antibodies against WNV and USUV. Seroprevalence for neutralizing antibodies against WNV and USUV was 84.1% and 10.8%, respectively. Management and age were significantly associated with WNV and USUV seropositivity. This is the first report describing exposure of horses in Israel to USUV, which indicates that this zoonotic pathogen should be included in the differential diagnosis list of neuroinvasive disease in this country.

## 1. Introduction

West Nile virus (WNV) is an ssRNA virus of the *Flaviviridae* family, *Flavivirus* genus and is the causative agent of West Nile fever (WNF). The virus is a mosquito-transmitted pathogen affecting various species of birds, as well as horses and humans [1,2,3]. While migratory and domestic avian species serve as natural virus reservoir, humans and horses are considered dead-end hosts [1,3,4]. In nature, the virus is maintained in mosquito–bird–mosquito transmission cycles [5]. WNV infection in mammals is mostly subclinical and, in some cases, may either cause flu-like symptoms, such as fever, headache (in humans) and malaise, or neuroinvasive disease that may manifest in meningoencephalitis or flaccid paralysis. Severe neuroinvasive disease in both horses and humans may lead to death and, in humans, affect mostly elderly peoples and immunocompromised individuals [1,6]. WNV was identified in all continents except Antarctica, where its major vector is absent [7]. In Australia, a particular indigenous strain of WNV, Kunjin virus (WNV_KUN_), exists [8]. WNV is currently recognized as one of the most widespread arboviruses [7,9].

Usutu virus (USUV) is an arbovirus of the *Flaviviridae* family, first isolated in Africa in 1959 [10]. The virus is transmitted by mosquito vectors, mainly *Culex* spp. The natural reservoir of the virus are birds, while other species such as humans and horses may serve as dead-end hosts [11,12,13]. Since 1996, infection with USUV was found in European countries including Italy, Croatia, Poland, Austria, and Germany, where mortality in birds, morbidity in humans, and serologic exposure in horses were reported [11,14,15,16]. Infection in horses may go unnoticed and clinical disease is considered mild [17,18]. Like WNV, although less common, USUV may cause neuroinvasive disease in humans [19]. Data regarding the presence of USUV in the Middle East is limited and, although information regarding WNV is available and the relevant vectors are prevalent, the prevalence of this virus was not reported in countries in this region.

Since Israel is located on a central pathway for birds migrating between Africa, Europe, and Asia, and since *Culex* spp. are the most common type of mosquitoes in Israel, there is high potential for exposure of humans and horses to arbovirus diseases. WNV is endemic in Israel, with human and equine clinical cases reported annually, and is monitored by active surveillance and screening of mosquitoes from various locations throughout the country [3,13,20]. Over the last decade, the reported seroprevalence of WNV in horses in Israel was over 80% [9,21]. USUV was isolated for the first time in Israel in 2014–2015 from six positive pools of mosquitoes in the north of the country [13]. However, no human or equine clinical cases of USUV have been reported in Israel. Due to their management, horses are exposed to flying insects and may develop immune response to USUV [11], and can therefore be potentially used as sentinels to USUV. The aim of this study was to assess horses’ exposure to USUV, to re-assess their exposure to WNV, and to determine the association between the two.

## 2. Materials and Methods

### 2.1. Sample Collection

A cross-sectional study was conducted from September to November 2018, in 28 farms. Farms were located throughout Israel according to the estimated geographical distribution of horse farms in the country. Serum was sampled from healthy horses. Horses that were vaccinated against WNV were excluded from the study. Blood samples were collected via jugular venipuncture into plain vacutainers, and sera were harvested after centrifuging at 3000× *g* for 10 min at room temperature. Serum samples were stored in −20 °C until analysis. Sample collection was performed under the horse owners’ consent, and the study was approved by the Internal Research Committee of the Koret School of Veterinary Medicine–Veterinary Teaching Hospital (KSVM-VTH/08_2017).

### 2.2. Virus Neutralization Test

Vero cells were grown in Eagle’s medium with 10% fetal bovine serum (Biological Industries, Cromwell, CT, USA), 1% antibiotic solution (PSA), including penicillin streptomycin, and amphotericin (Biological Industries) and 0.4% L-glutamine (Biological Industries). The cells were maintained in a humidified atmosphere at 37 °C in 5% CO_2_. For virus neutralization testing (VNT), 4 days old Vero cell culture were chemically scrapped with EDTA trypsin 0.2% (Biological Industries) and seeded in 96-well flat sterile microplates (Greiner Bio-One, Frickenhausen, Germany). 

For the WNV VNT, twofold dilutions from 1:2 to 1:512 of 50 μL of each serum sample (in duplicates) were made in 96-well plates (96 wells round bottom (U) microplates, Greiner Bio-One International) in 1% PSA minimal essential medium (MEM) (according to the standard of the World Reference Laboratory for Foot-and-Mouth Disease (WRL), Pirbright, UK). One hundred units (100 U) (10^2^ TCID_50_ (Median Tissue Culture Infectious Dose)/50 μL) of the challenge virus WN98 (GenBank AY033388) [22] were added to each well of serum dilutions’ plates. These plates were then allowed to stand for 30 min at 37 °C 5% CO_2_. After incubation, 100 μL of the Serum–Ag mixture was added to each well in the 96-well microplates containing freshly seeded Vero cells. The plates were placed in a 37 °C incubator (5% CO_2_) for 4 days, with daily microscopic examination for cytopathic effect (CPE). The neutralizing antibody titer of the serum was calculated as the highest dilution at which complete neutralization of CPE was observed. Samples with antibody titers of 1:16 or higher were considered positive.

For the USUV VNT assay, Usutu virus isolate that was received from the Israeli central virology laboratory, was grown and titered on Vero cells. USUV VNT was calibrated using positive control serum sample (from rabbits—provided by Martin Groschup and Utte Ziegler, Institute of Novel and Emerging Infectious Diseases (INNT), Friedrich Loeffler Institute, Greifswald, Germany) and 29 horse sera from 2017, which were tested negative for antibodies against WNV. In short, serum samples were diluted in serial order (1:4–1:512) in duplicates within 96-well plates using MEM medium with 1% antibiotics (ATB). Like in the WNV VNT protocol 100 U (10^2^ TCID_50_/50 μL) of USUV were divided to each well and plates were incubated in 37 °C for 30 min. One hundred µL of freshly passaged Vero cells were divided into 96-well cell culture plates. After incubation, 100 µL of each duplicated sample dilutions were added to each well of the Vero cells and incubated in 37 °C, 5% CO_2_. All plates were observed daily for CPE. The neutralizing antibody titer of the serum was calculated as the highest dilution at which complete neutralization of CPE was observed. Samples with antibody titers of 1:16 or higher were considered positive.

### 2.3. Statistical Analysis 

Risk factors associated with exposure to either virus (sampling location and animal sex) were assessed by using the two-sided Fisher’s exact test, and odds ratios were calculated. Association between these parameters and antibody titers were evaluated using the Mann–Whitney U test. The analysis was performed, using SPSS v22.0 and Win Pepi v11.43 statistical softwares. Statistical significance was set as *p* < 0.05.

## 3. Results

### 3.1. Study Population

A total of 327 horses were sampled at 28 farms, between four and 32 horses were sampled at each farm. Almost half of the horses were mixed breeds (152, 47%) and others were of various breeds including Quarter horses (66, 20%), Arabians (45, 13.8%), Ponies (19, 6%), Warmbloods (13, 4%), and Tennessee Walking horses (12, 4%). The population included 157 mares (48%), 163 geldings (50%), and seven stallions (2%). One hundred and thirty-eight horses were kept in stalls (42%), 125 were turned out in paddocks (38%), and 64 were kept in pasture (20%).

### 3.2. WNV Seroprevalence

WNV seroprevalence in the study population was 84.1% (275/327 horses). Antibody titers of positive samples ranged between 1:16 and 1:512 (median = 1:128, 50% interquartile range (IQR): 1:64–1:384) (Supplementary Table S1). Housing management was significantly associated with exposure to WNV, with horses kept in pasture having lower seroprevalence than horses kept in stalls or paddocks (odds ratio (OR) = 0.42, 95% confidence interval (CI): 0.22–0.84, *p* = 0.013). Seroprevalence was also associated with age (U-test *p* < 0.001). The mean age of seropositive animals (12.2 years, SDV (standard deviation) = 5.9, *n* = 273) was higher than the mean age of seronegative animals (8.6 years, SDV = 6, *n* = 50).

Of the 57 horses that have been previously sampled in 2015 [21], six were negative for WNV using cELISA and remained negative in 2018 using VNT. Of the 51 horses that tested positive in 2015, 41 (80.4%) remained positive, while 10 tested negative in 2018. Titers for neutralizing antibodies for these 2015 ELISA positive samples were negative or very low in WNV VNT test. 

### 3.3. USUV Seroprevalence

Fifty WNV-seronegative and 135 WNV-seropositive horses were tested for serological exposure to USUV. The seroprevalence of USUV was 10.8% (20/185 horses) and did not differ significantly between WNV-negative (4/50, 8%) and WNV-positive (16/135, 11.9%) (*p* = 0.454) horses. USUV seroprevalence was higher in the north of Israel, in mixed breed horses and in horses kept in pasture (Table 1). The mean age of seropositive horses was significantly higher than the mean age of seronegative horses (15.2 and 10.6 years, respectively, *p* = 0.006). In the multivariable analysis, pasture (*p* = 0.031) and age (*p* < 0.001) remained significantly associated with exposure. Antibody titers of positive samples ranged between 1:16 and 1:32 (median = 1:16, Supplementary Table S1).

### 3.4. WNV and USUV Co-Exposure

Antibodies against both viruses were detected in 16 of 185 horses (8.6%). Horses from the north of Israel and horses kept in pasture had higher co-exposure rates (Table 1). The mean age of seropositive horses to both USUV and WNV was significantly higher than the mean age of seronegative horses (16.2 and 10.6 years, respectively, *p* = 0.002). Age was the only significant factor in the multivariable model (*p* < 0.001).

## 4. Discussion

Seroprevalence of WNV neutralizing antibodies in horses in Israel in 2018 was similar to the previously reported prevalence in 2015 [21]. Sampling of horses in 2018 followed summer outbreak of WNV in that year that was accompanied by increased morbidity and mortality in wild avian species in Israel [23]. However, morbidity and mortality in the horse population in Israel were not exceptionally high in 2018 (diagnostic documentation—unpublished data and personal communication). The high exposure of horses to WNV and an increase in WNV vaccination practice may explain the relative low rate of clinical infection with WNV in the horse population in Israel. 

Similar to 2015 [21], horses kept in pasture had lower seroprevalence of WNV than horses kept in stalls or paddocks, indicating relatively lower exposure in pasture horses, possibly due to relatively lower density of the vector in dry and well ventilated locations. This is in contrast to what was found in a study in Tunisia where equids (horses, mules, and donkeys) kept outside had a significant increased risk of being seropositive compared with those kept inside stable [24]. In Israel, most of the pasture areas are located in the northern parts of the country, which differ from other geographical areas in the country and, therefore, these results may result from other environmental variables in different geographical locations. It is also possible that the positive association between exposure to USUV and pasture may be contributed to the reported USUV positive mosquitoes’ pools that were reported from the north (2014–2015) [13]. 

As in most serological studies, age was associated with exposure of horses to either WNV, USUV, or both, as the chance for exposure and possibly re-exposure to infected mosquito may increases with age. Interestingly, all six seronegative horses that were tested (WNV VNT) both in 2015 and 2018 remained negative. No significant correlation was found with coat color, geographic location, age, or management practice in these horses. We are uncertain whether these horses are less likely to be bitten by mosquitoes or that their immunologic response is different to this viral infection. It is conceivable that individual immune status, and/or chemical composition of the skin and coat may contribute to their relative resistance to exposure or infection that is characterized by neutralizing antibody production.

While USUV RNA was detected in mosquito pools in the north of Israel in 2014–2015 [13], no clinical infection was reported in humans or animals in Israel to date. Infection with USUV is considered mild and mostly self-limiting, however neuro-invasiveness has been reported in birds and humans [11,12,17,19,25,26,27]. Therefore, it is possible that this infection occurred, but was not diagnosed in Israel. Seroprevalence to USUV in horses in Israel was significantly lower than seroprevalence to WNV, probably due to relatively limited circulation of this virus in mosquitoes in Israel. 

Titers of neutralizing antibodies to USUV were relatively low (≤1:32) and it is possible that those are fading titers from an old infection potentially related to the reported circulation of the virus in mosquitoes in Israel in 2014–2015 [13]. It is also possible that horses do not show a strong immune response as was previously suggested [17]. The higher titers of neutralizing antibodies to WNV may be attributed to its established occurrence and bird migration dependent circulation in mosquitoes in Israel, and to the potential of frequent re-exposure. 

In samples that were positive to neutralizing antibodies against both WNV and USUV, titers of the first were significantly higher (over 4-fold). Differences in titer levels may be due to the varying time that passed since the exposure and to differences in viral infective dose. In these cases, cross reactivity was considered; however, since the majority of WNV positive sampled were USUV negative, cross protection between these two Flaviviruses does not necessarily occur even when titers are high. 

When assessing the seroconversion of horses to WNV between 2015 and 2018, it is important to take into consideration the differences in the methodology and interpretation of the results. While WNV VNT tests for neutralization of the WNV (inhibition of CPE in sensitive cells), the ELISA test detects specific antibodies that are produced against WNV (patent protected). Positive WNV IgG ELISA assay indicates exposure to WNV; however, WNV VNT assay indicates sufficient neutralizing response against WNV. Thus, agreement is not necessarily optimal. Moreover, immunologic reaction may be different between individuals and the result of the serology analysis in these two methods does not always correlate. The results of this study demonstrate that horses can be used for detection of WNV and USUV circulation in Israel. The varying antibody titers identified here highlight the importance of continuous surveillance of these viruses, in order to identify trends in their circulation before increased morbidity and mortality can occur. 

## Figures and Tables

**Table 1:** Risk factors associated with exposure to West Nile virus (WNV) and Usutu virus (USUV) (*N*: number of horses, OR: odd ration, CI: confidence interval, *p*: *p*-value, Ref: reference group for groups of the same category, i.e., location, breed, etc.).

	*N*	USUV-Positive (%)	OR (95% CI)	*p*	Co-Infection (%)	OR (95% CI)	*p*
North	63	15 (23.8)	17.8 (2.5–762.7)	<0.001	11 (17.5)	12.06 (1.62–527.9)	0.005
Center	64	4 (6.3)	3.8 (0.36–190.4)	0.368	4 (6.3)	3.8 (0.36–190.4)	0.368
South	58	1 (1.7)	ref		1 (1.7)	ref	
Mixed	77	13 (16.8)	2.93 (1.02–9.1)	0.031	10 (13)	2.54 (0.79–8.9)	0.11
Pure	108	7 (6.5)	ref		6 (5.6)	ref	
Mare	95	12 (12.6)	1.4 (0.5–4.2)	0.636	10 (10.5)	1.57 (0.49–5.49)	0.443
Stallion	4	0	0 (0–17)	1	0	0 (0–24.46)	1
Gelding	86	8 (9.3)	ref		6 (7)	ref	
Pasture	36	12 (33.3)	21 (4.1–199.6)	<0.001	8 (22.2)	12 (2.17–119.6)	<0.001
Paddock	63	6 (9.5)	4.42 (0.75–45.84)	0.071	6 (9.5)	4.42 (0.75–45.84)	0.071
Stall	86	2 (2.3)	ref		2 (2.3)	ref	

## Supplementary Materials

The following are available online at https://www.mdpi.com/1999-4915/12/10/1099/s1, Table S1: Titers of neutralizing antibodies for WNV and USUV.
