# Supplementary material for: Exposure of Horses in Israel to West Nile Virus and Usutu Virus
Source: Viruses. 2020 Sep 28;12(10):1099. doi: 10.3390/v12101099 (PMC7650752; doi:10.3390/v12101099)
Supplement: Supplementary file 1 [file viruses-12-01099-s001.zip › viruses-909259-supplementary.pdf]

viruses

Supplementary

Exposure of Horses in Israel to West Nile Virus and Usutu Virus

Gili Schvartz [1,2], Sharon Tirosh-Levy [1], Oran Erester [3], Roni Shenhar [1], Hadas Levy [1], Barbara Bazanow [4], Boris Gelman [2] and Amir Steinman [1,*]

[1] Koret School of Veterinary Medicine, the Hebrew University of Jerusalem, Rehovot 7610001, Israel; giliun@gmail.com (G.S.); sharontirosh@gmail.com (S.T.-L.); rony.shenhar@mail.huji.ac.il (R.S.); hadasylevy@gmail.com (H.L.)
[2] Department of Virology, Kimron Veterinary Institute, Bet Dagan 50200, Israel; borisg@moag.gov.il
[3] Central Virology Laboratory, Haim Sheba Medical Center, Ministry of Health, Ramat-Gan 5265601, Israel; Oran.Erster@sheba.health.gov.il
[4] Department of Pathology, Wrocław University of Environmental and Life Sciences, 50-375 Wroclaw, Poland; barbara.bazanow@upwr.edu.pl
* Correspondence: amirst@savion.huji.ac.il; Tel.: +972-3-9688544

Abstract: West Nile virus (WNV) and Usutu virus (USUV) are arboviruses transmitted by mosquito vectors. Whereas WNV is endemic in Israel, the Middle East, Europe, and in the Americas, data regarding the prevalence of USUV in the Middle East is limited. While both viruses share similar reservoirs and vectors, exposure of horses in the area to USUV have never been assessed. The aim of this study was to estimate the seroprevalence and co-exposure of WNV and USUV in horses in Israel. A total of 327 serum samples from healthy unvaccinated horses in Israel collected in 2018 were tested for neutralizing antibodies against WNV and USUV. Seroprevalence for neutralizing antibodies against WNV and USUV was 84.1% and 10.8%, respectively. Management and age were significantly associated with WNV and USUV seropositivity. This is the first report describing exposure of horses in Israel to USUV, which indicates that this zoonotic pathogen should be included in the differential diagnosis list of neuroinvasive disease in this country.

Keywords: West Nile virus; Usutu virus; horse; Israel

Supplementary Table S1. Titers of neutralizing antibodies for WNV and USUV.

**Last dilution where CPE was prevented**	**WNV *N* (%)**	**USUV *N* (%)**
0	33 (10.1)	68 (20.8)
1:2	1 (0.3)	46 (24.9)
1:4	3 (0.9)	15 (4.6)
1:6	2 (0,6)	20 (6.1)
1:8	7 (2.1)	16 (4.9)
1:12	6 (1.8)	12 (3.7)
1:16	14 (4.3)	5 (1.5)
1:24	12 (3.7)	3 (0.9)
1:32	16 (4.9)	
1:48	21 (6.4)	
1:64	24 (7.3)	
1:96	32 (9.8)	
1:128	19 (5.8)	
1:192	14 (4.3)	
1:256	27 (8.3)	
1:384	34 (10.4)	
1:512	62 (19.0)	
**Total *N***	**327**	**185**
